# Evidences on the Use of Hypofractionation in Postoperative/Salvage Radiotherapy for Prostate Cancer: Systematic Review of the Literature and Recent Developments

**DOI:** 10.3390/cancers16244227

**Published:** 2024-12-18

**Authors:** Camille Roukoz, Amina Lazrek, Lilia Bardoscia, Giuseppe Rubini, Chieh-Min Liu, Anne-Agathe Serre, Angela Sardaro, Dino Rubini, Sarah Houabes, Cecile Laude, Salvatore Cozzi

**Affiliations:** 1 Radiation Oncology Department, Centre Leon Berard, 69373 Lyon, France; anne-agathe.serre@lyon.unicancer.fr (A.-A.S.); cecile.laude@lyon.unicancer.fr (C.L.); 2 Radiation Oncology Unit, International University Hospital Cheikh Zaid, Rabat 10000, Morocco; amina.lazrek@lyon.unicancer.fr; 3 Radiation Oncology Unit, S. Luca Hospital, Healthcare Company Tuscany Nord Ovest, 55100 Lucca, Italy; lilia.bardoscia@uslnordovest.toscana.it; 4 Interdisciplinary Department of Medicine, Section of Nuclear Medicine, University of Bari, 70124 Bari, Italy; giuseppe.rubini@uniba.it; 5 Department of Radiation Oncology, Proton and Radiation Therapy Center, Kaohsiung Chang Gung Memorial Hospital, Kaohsiung City 83062, Taiwan; mmmin@cgmh.org.tw; 6 Interdisciplinary Department of Medicine, Section of Radiology and Radiation Oncology, University of Bari “Aldo Moro”, 70124 Bari, Italy; angela.sardaro@uniba.it (A.S.); dino.rubini@libero.it (D.R.); 7 Radiation Oncology Unit, Portes de Provence Hospital Groupe, 26200 Montélimar, France; sarah.houabes@ghpp.fr

**Keywords:** prostatic bed radiotherapy, hypofractionated radiotherapy, hypofractionated post-operative radiotherapy, salvage radiotherapy

## Abstract

Background: Radical prostatectomy (RP) is one possible curative treatment for localized prostate cancer. Despite that, up to 40% of patients will later relapse. Currently, post-operative radiotherapy (PORT) courses deliver 1.8–2 Gy daily to reach a total dose ranging between 64 and 74 Gy, completed in 7–8 weeks. Several articles reported encouraging data in terms of the effectiveness and the related toxicities using hypofractionation schedules. The objective of the present systematic review was to evaluate the clinical outcomes and toxicity of the use of hypofractionation in adjuvant/salvage prostate cancer treatments. Methods: Medline was searched via PubMed and Scopus from inception to July 2024 to retrieve studies on hypofractionation in adjuvant/salvage prostate cancer treatments. This study was conducted under PRISMA guidelines. Results: A total of 139 articles were identified from the initial search. Subsequently, the 139 studies were reviewed by title and abstract. Ninety-five studies were excluded due to being either abstracts or articles not available in English. In the second step, the full texts of 44 studies were reviewed. Eleven studies were excluded for being reviews, study protocols, or focused on SBRT treatments. Finally, 33 studies were included in our analysis, with a total number of 4269 patients. Of the 33 selected studies, 20 were retrospective trials and 11 were phase I/II prospective trials, while 2 studies were prospective phase III trials. The follow-up ranged from 18 to 217 months. Failure-free survival, for those with the longer follow-up, ranged between 85% and 91% at 3 years, 47 and 78.6% at 5 years and 51.5% at 10 years. Genitourinary (GU) and gastrointestinal acute toxicity was mild to moderate with similar rates across the normofractionated and hypofractionated groups. Acute grade-3 GU toxicity events were unusual, occurring in less than 4% of the cases overall. Conclusion: The present study is the first systematic review of the literature that includes the first two randomized phase III studies published in the literature. Hypofractionated treatment has been shown to be safe, effective, with moderate toxicity and not inferior to conventional RT, with good biochemical control rates.

## Simple Summary

The objective of the present systematic review was to evaluate the clinical outcomes and toxicity of the use of hypofractionation in adjuvant/salvage prostate cancer treatments. A total of 139 studies were identified and, finally, 33 studies were included in our analysis, with a total number of 4269 patients. Biochemical relapse-free survival (bRFS) ranged between 85% and 91% at 3 years, and between 47% and 78.6% at 5 years. Genitourinary (GU) and gastrointestinal (GI) acute toxicity was mild to moderate with comparable rates across the studies. Although some doubts remain on late toxic effects, hypofractionated treatment has been shown to be safe, effective, with moderate toxicity and not inferior to conventional RT, with good biochemical control rates.

## 1. Introduction

Prostate cancer (PCa) is the most common cancer in the male population [1]. Radical prostatectomy (RP) is one possible treatment choice for localized prostate cancer, notwithstanding that up to 40% of patients will later have a recurrence [2,3]. After RP, post-operative radiotherapy (PORT) may be administered for adverse pathologic features (i.e., seminal vesicle invasion (cT3b), extra prostatic extension, positive surgical margins). In fact, adjuvant radiotherapy (RT) was shown to improve overall survival (OS) in one randomized controlled trial and biochemical control in two others [2,3,4]. For men with biochemical recurrence following RP, salvage RT has been shown to improve prostate cancer specific survival compared to observation [5]. Based on the radiobiological properties of PCa, as a cancer very sensitive to higher doses per fraction, the growth of modern RT techniques led to the current extent of moderate and extreme hypofractionated treatments for the non-surgical patient [6,7,8,9,10,11]. Recently, the RADICALS trial, a phase III trial comparing adjuvant versus early salvage radiotherapy along with the inclusion of androgen deprivation therapy (ADT), do not support PORT, as adjuvant radiotherapy increases the risk of urinary side effects. An observation policy with salvage RT for PSA biochemical progression should be the current standard of care [12]. Currently, PORT courses deliver 1.8–2 Gy daily to reach a total dose between 64 and 74 Gy in 7–8 weeks. In the post-operative setting, the role of hypofractionated RT is not routinary. Several retrospective studies and phase I/II trials report encouraging biochemical control following hypofractionated PORT [13]. The objective of the present systematic review was to evaluate the clinical outcomes, in terms of overall local control and toxicity, of the use of hypofractionation in adjuvant/salvage prostate cancer treatments.

## 2. Materials and Methods

### 2.1. Object

This study aimed to investigate the clinical outcomes and the toxicity profile of hypofractionated RT in the management of adjuvant/salvage PCa.

This study was designed following the Preferred Reporting Items for Systematic Reviews and Meta-Analyses (PRISMA) guidelines [14].

### 2.2. Search Strategy

To retrieve the studies, Medline was searched via PubMed from inception to May 2024. The gray literature was also reviewed through a manual search of Google Scholar. The relevant keywords were obtained from reviewing the literature, and the electronic databases were searched using the following: adjuvant radiotherapy, salvage radiotherapy, prostate cancer, hypofractionation, radiotherapy on the prostatectomy bed, radiotherapy after prostatectomy, radiotherapy following prostatectomy, post prostatectomy radiation therapy, hypofractionated postoperative radiotherapy for prostate cancer, hypofractionated salvage radiotherapy for post prostatectomy biochemical recurrence.

### 2.3. Eligibility Criteria

The studies having both the inclusion and exclusion criteria were considered for data extraction.

Inclusion criteria:

(1) Articles written in English.
(2) Studies on human subjects.
(3) Original articles.

Exclusion criteria:

(1) Non-English articles.
(2) In vivo or in vitro studies.
(3) Review articles, conference abstracts, letters to editor, book chapters, or case reports.

### 2.4. Study Selection

Two reviewers independently managed the study selection process in two steps, the title/abstract and full-text assessment processes. The studies retrieved from a search of databases were examined for the study selection process. First, the title and abstract were assessed and the articles pertinent to the study object were taken for consideration in the full-text assessment. Second, in the full-text assessment process, the papers fully fitting our eligibility criteria were considered for data extraction.

### 2.5. Data Extraction

Two reviewers independently extracted the data from the final included articles. The information of articles was extracted as follows: first author name, year of publication, total dose/dose for fraction, outcomes, toxicity, dose constraints. The extracted data were checked by a third reviewer.

## 3. Results

**Figure 1:**
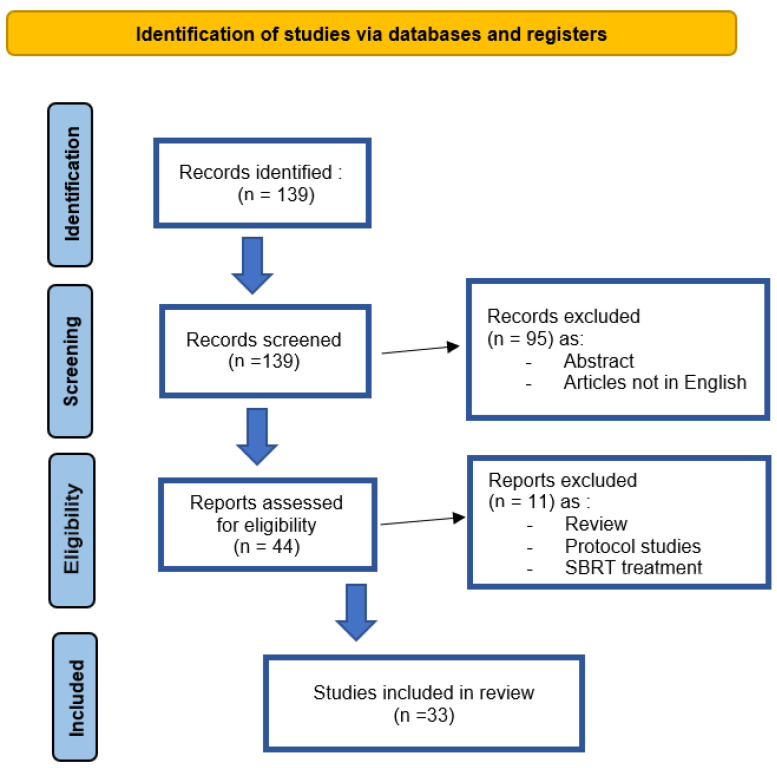
The PRISMA flowchart.

According to the purpose of this study, 139 articles were identified that had been published from 2008 to 2024. The initial search identified 67 results on PubMed and 72 results on Scopus. Subsequently, the 139 studies were reviewed by title and abstract. In the second step, the full texts of 44 studies were reviewed. Finally, 33 studies were included in our analysis, with a total number of 4269 patients. Figure 1 shows the flowchart for screening the eligible studies.

Of the 33 selected studies, 20 were retrospective and 11 were phase I/II prospective trials, while 2 studies were prospective phase III trials. The selected studies were published between 2008 and 2024 (Table 1). The follow-up ranged from 18 to 217 months. The different fractionation schedules used in the corresponding studies are reported in Table 1. The total dose to the prostate bed ranged from 51 Gy in 14 fractions to 71.4 Gy in 28 fractions. The normalized total dose in 2 Gy/fraction (NTD2Gy), assuming an a/b value of 3 Gy for late toxicity, ranged from 58 Gy to 80 Gy. Most studies used a moderate fractionation schedule in 20 or more sessions. Only 6 studies had a slightly more hypofractionated schedule with less than 20 fractions [15,16,17,18,19]. All but six studies using 3D techniques used complex techniques including intensity-modulated RT (IMRT), volumetric modulated-arc RT (VMAT) or helical tomotherapy [15,20,21,22,23]. The RT technique employed was not mentioned in one article. Pretreatment rectal cleaning and bladder filling protocols were used in all patients. For most patients, image-guided radiotherapy (IGRT) techniques with daily CBCTs or kV/MV images were performed. Some investigators used an endorectal balloon for intra-fractional immobilization, whereas Gladwish et al. implanted three fiducial markers in the prostate bed to aid with daily imaging guidance [17,24,25].

Data from patients treated with hyaluronic acid with the aim of preserving the rectum were reported by Nicosia et al. [26].

Combining ADT with EBRT was not usually recorded, but when it was, the time and duration (concomitant, adjuvant or neo-adjuvant) varied greatly between series.

Data regarding quality of life (QoL) using two validated international QoL questionnaires, the EPIC (Expanded Prostate Cancer Index Composite) and the EORTC QLQ-C30, were reported in five studies; in three [12,16,27], the scores did not change after the hypofractionated treatment, while sexual changes were reported in 22.2 % and 51 patients in the Gladwish and Fersino studies, respectively [17,28].

**Table 1:** Treatment characteristics and outcomes.

Study	N of PT	FUP (M)	Total Dose/Dose for Fraction/Number of Fractions	NTD2GY	Treatment Technique	Outcomes	QoL
Wong et al. Retrospective (2008) [25]	50	24	65 Gy/2.5G y/26fr	74.3 Gy	IMRT-HT	2yr-bPFS: 72.9% 1yr-OS: 96% 1yr-DFS: 2%	NR
Kruser et al. Retrospective (2011) [24]	108	32.4	65 Gy/2.5 Gy/26fr	74.3 Gy	IMRT-HT	4yr-bPFS: 67% 4yr-OS: 99% 4yr-DFS: 2.7%	NR
Koukourakis et al. Phase II (2012) [15]	48	41	Pelvis: 38.6 Gy/2.7 Gy/14fr Prostatic bed (SIB): 51 Gy/3.6 Gy/14 fr	65.3–78.1 Gy	3D-CRT	3yr-bPFS: 85.4%	NR
Ippolito et al. Phase II (2013) [29]	25	19	56.8 Gy/2.27 Gy/25 fr 59.7 Gy/2.39 Gy/25fr 61.25 Gy/2.45 Gy/25fr 62.5 Gy/2.5 Gy/25fr	61.1 Gy 66.4 Gy 69.1 Gy 71.4 Gy	IMRT	NR	NR
Alongi et al. Retrospective (2013) [30]	84	24.1	70–71.4 Gy/2.5–2.55 Gy/28Fr	80 Gy	IMRT	NR	NR
Massaccesi et al. Phase II (2013) [31]	43	24	62.5 Gy/2.5 Gy/25Fr	68.75 Gy	IMRT	NR	NR
Cozzarini C. Retrospective (2014) [32]	247	98	65.8 Gy/2.35 Gy/28 fr 71.4 Gy/2.5 Gy/28 fr 58 Gy/2.5 Gy/20 fr	65.8 Gy 71.4 Gy 58 Gy	IMRT-HT	NR	NR
Katayama et al. Phase II (2014) [16]	39	NR	54 Gy/3 Gy/18fr	69.4 Gy	IMRT-HT	NR	General stability throughout treatment
Gladwish et al. Phase I/II (2015) [17]	30	24	51 Gy/3 Gy/17fr	66 Gy	IMRT	2yr-bPFS: 90%	Sexual change in 22.2 % of pt
Lewis et al. Retrospective (2016) [33]	56	48	65 Gy/2.5 Gy/26fr	71.4 Gy	IMRT	4yr-bPFS: 75% 4yr-OS: 96%	NR
Fersino et al. Retrospective (2017) [28]	125	18	66 Gy(65.5–71.4)/2.3 Gy(2.2–2.4)/20–30fr	74 Gy	VMAT	3yr-bPFS: 94% 3yr-bPFS:77% 3yr-OS: 96%	51 patients were noted to have erectile dysfunction
Macchia et al. Phase II (2017) [34]	124	60	62.5 Gy/2.5 Gy/25fr	71.4 Gy	IMRT	3yr-bPFS: 86.5% 3yr-OS: 100% 3yr-DFS: 98%	NR
Cuccia et al. Retrospective (2018) [35]	75	30	63.8 Gy/2.2 Gy/29fr	67.4 Gy	IMRT-HT	3yr-bPFS: 73%	NR
Tramacere et al. Retrospective (2018) [36]	69	54.7	62.5 Gy/2.5 Gy/25fr	71.4 Gy	IMRT	5yr-bPFS: 66.7% 5yr-OS: 91.1% 5yr-DFS: 84.6%	NR
Tandberg et al. Retrospective (2018) [27]	167	38.6	65 Gy/2.5 Gy/26 fr	74.3 Gy	IMRT/VMAT	4yr-bPFS: 78.4% 4yr-OS: 94.3% 4yr-DFS: 95.4%	No significant difference in quality of life scores
Barra et al. Retrospective (2018) [37]	64	72	62.5 Gy/2.5 Gy/25 fr	71.4 Gy	VMAT	5yr-OS: 98 %	NR
Saldi et al. Phase II (2019) [38]	112	NR	65.25 Gy/2.25 Gy/29fr 72–74.25 Gy/2.25 Gy/32–33 fr	NR	IMRT-HT	NR	NR
Ishikawa et al. Retrospective (2020) [20]	38	62	60 Gy/3 Gy/20 Fr	77.14 Gy	3D-CRT	5yr-PFS: 47.4% 5yr-OS: 97.1% 5yr-DFS: 9.8%	NR
Leite et al. Phase II (2021) [18]	61	16	51 Gy/3.4 Gy/15fr	NR	IMRT	1yr-PFS: 95.1%	NR
Chin et al. Retrospective (2020) [21]	112	120	52.5 Gy/2.62 Gy/20fr	61.9 Gy	3D-CRT	10yr-PFS: 51.5% 10yr-OS: 75% 10yr-DFS: 16%	NR
Wages et al. Phase I/II (2021) [19]	32	42.3	56.6 Gy/2.8 Gy/20fr 50.4 Gy/3.36 Gy/15fr 42.6 Gy/4.26/10fr	NR	IMRT	NR	NR
Valero et al. Retrospective (2021) [39]	113	29	62.5 Gy/2.5 Gy/25fr	71.4 Gy	IMRT/VMAT	3yr-PFS: 91.1% 1yr-DFS: 8.8%	NR
Franzese et al. Retrospective (2021) [40]	181	54.5	70 Gy (65–74.2 Gy)/2.55 (2.3–2.8)/(25–28 fr)	80 Gy	VMAT	NR	NR
Murgic et al. Retrospective (2021) [22]	147	67	52.5 Gy/2.62 Gy/20fr	59 Gy	3D-CRT	5yr-PFS: 76.9%	NR
Viani et al. Prospective (2022) [41]	412	31	52.5 Gy/2.625 Gy/20fr	70 Gy	3D-CRT/IMRT	3yr-bPFS: 93%	NR
Ferrera et al. Retrospective (2022) [42]	129	43	61.6 Gy/2.12 Gy/29 fr 65.25 Gy/2.25 Gy/29fr	NR	IMRT-HT	2yr-PFS: 78.7% 1yr-OS: 98.4% 1yr-DFS: 1.6%	NR
Nicosia, L et al. Retrospective (2022) [26]	305	NR	Adjuvant RT: 66 Gy/2.2 Gy/30fr Salvage RT: 67.5 Gy/2.25 Gy/30fr Pelvis: 51 to 52.5 Gy	NR	VMAT	NR	NR
Moll et al. Retrospective (2022) [43]	283	72	62.5–63.75 Gy/2.5–2.55 Gy	NR	3D-CRT/IMRT	1yr-PFS: 77% 5yr-OS: 95%	NR
Dubinsky et al. Prospective (2023) [44]	100	61	52.8 Gy/3.3 Gy/16fr	NR	IMRT/VMAT	5yr-PFS: 78.6% 5yr-DFS: 4.3%	NR
Buwenge et al. Retrospective (2023) [45]	381		2.2 to 2.6 Gy per fraction	66.2–78 Gy	IMRT/VMAT/3D-CRT	NR	NR
Petersen et al. Phase III (2023) [46]	217	58	52.5 Gy/2.62 Gy/20 fr	61.8 Gy	IMRT	7yr-PFS: 95% 7yr-OS: 95% 1yr-DFS: 5%	Patients reported similar physical and mental health scores at baseline and at 1 year
Castelluccia et al. Retrospective (2024) [23]	110	103	62.5 Gy /2.5 Gy/25 fr	70 Gy	3D-CRT	10yr-PFS: 53.3% 10yr-OS: 77.3% 10yr-DFS: 23.3%	NR
Buyyounouski et al. Phase III (2024) [47]	144	24	62.5 Gy/2.5 Gy/25 fr	NR	IMRT/VMAT/IRM guided RT	2yr-PFS: 72.9% 2yr-OS: NR 2yr-DFS: NR	NR

Abbreviations: N: number; pt: patients; FUP: follow-up; m: months; NTD2Gy: normalized total dose in 2 Gy/fraction; QoL: quality of life; Gy: Gray; NR: not reported, y: year; bPFS: biochemical progression-free survival; OS: overall survival; DFS: disease-free survival; IMRT: intensity-modulated radiation therapy; VMAT: volumetric modulated arc therapy; HT: helicoidal tomotherapy; 3D-CRT: Three-Dimensional Conformal Radiation Therapy.

Biochemical relapse-free survival (bRFS) rates were reported in the majority of studies presented and the results were comparable to those reported in the literature, with failure-free survival, for those with the longer follow-up, ranging between 85% and 91% at 3 years, 47 and 78.6% at 5 years [20,22,36,37,44] and 51.5% at 10 years [18,23]. Two phase III randomized controlled trials published their results regarding bRFS. The RADICALS trial, comparing adjuvant RT with salvage RT, used two different RT schemes: 66 Gy in 33 fractions or 52.5 Gy in 20 fractions [46]. The clinical results were not stratified according to the RT schedule. With a median follow-up of 7.8 years, Petersen et al. found a 5-year biochemical progression-free survival (bPFS) of 85% for those in the group receiving adjuvant RT and 88% for those in the salvage radiotherapy group (HR 1.10, 95% CI 0.81–1.49; *p* = 0.56). No statistically significant difference in metastasis-free survival (MFS) and OS between the adjuvant RT group and the salvage RT group were found. The NRG-GU003 trial compared two different RT fractionations in the post-operative setting: the HYPORT group received 62.5 Gy in 25 fractions and the COPORT received 66.6 Gy in 37 fractions. The bPFS was not significantly different at 2 years (HYPORT: 12%, COPORT: 8%; *p* = 0.28). The MFS and OS are not published yet. The 5-year OS ranged from 91 % to 98 %, while the 10-year OS ranged from 77.3% to 98% [47].

Genitourinary (GU) and gastrointestinal (GI) acute side effects were moderate with comparable rates across the series (Table 2). Indeed, grade-2 changes in urinary frequency, urethra stenosis or proctitis were reported in the majority of the selected studies. Acute grade-3 GU toxicity events were uncommon, with less than 4% of the cases overall. A single experience of acute G4 toxicity was reported in four studies [17,19,31,34]. In the RADICALS trial, the toxicity in the experimental arm was as follows: 2yr GU G1 and G2: 9%, six patients had G3 diarrhea, two patients grade G3 proctitis, two patients G3 cystitis, seven patients G3 hematuria and nine patients G3 urethral stenosis. After 2 years, one patient had G4 diarrhea, one patient G3 proctitis, one patient G3 cystitis, six patients G3 hematuria and five patients G3 urethral stenosis [46].

Although there were noticeable follow-up differences, late toxicity was reported in all but three series. The majority of series had moderate (grade 1–2) late toxicity, which included proctitis, changes in urgency, frequency, or frequency of urination, or episodes of macrohematuria. Nonetheless, Cozzarini et al. provided the most thorough and pertinent information regarding long-term GU toxicity [32]. In fact, hypofractionated helical tomotherapy (*n* = 247) or conventionally fractionated EBRT (*n* = 929), primarily using 3D conformal techniques (*n* = 657), were used to treat 1176 postoperative patients. The 5-year risk for grade-3 late GU toxicity was considerably higher in the hypofractionated group (18.1%) than in the patients treated with standard EBRT (6.9%) after a median follow-up time of 68 months (range, 54–81). Of the 115 patients with late grade-3 GU toxicity, 47 patients had grade-3 urinary incontinence, 30 patients received blood transfusions and/or hyperbaric oxygen therapy for severe hematuria and 68 patients needed surgery for urethral and/or bladder neck stenosis. Five patients suffered grade-4 GU toxicity and underwent salvage cystectomy. Lewis et al. reported the side effects in a cohort of 56 patients treated with 65 Gy in 2.5 Gy/fr. Fifteen out of 56 patients (27%) had a G3 hematuria, while two presented with late G3 rectal fistulas requiring surgical correction. In summary, severe toxicity was reported in nine articles [17,22,27,28,30,32,36,42,43], while in six studies it was not reported [19,20,26,29,40,41]. 

## 4. Discussion

RT is a milestone in the management of PCa with a definitive intent even in the salvage/adjuvant setting. Emerging data have established RT as a useful therapeutic option also for oligometastatic and oligorecurrent/oligoprogressive disease, rare histologies, or in combination with new drugs available for hormone-sensitive and castration-resistant PCa [48,49,50].

Advances in RT planning and delivery methods have improved treatment precision in recent years, leading to the use of hypofractionated regimens in various oncological situations, such as stereotactic body RT (SBRT) [51,52,53,54,55,56,57].

Furthermore, randomized trials have validated hypofractionated EBRT as a safe and efficient treatment option for non-metastasized PCa, in contrast to normofractionated regimens [7,8,9,58]. These data are connected with the low a/b value of the prostate cancer, between 1–2 Gy, that is lower than the surrounding organs at risk [59,60,61]. So, delivering fewer but higher doses per fraction (i.e., >2.5 Gy) compared to conventional schedules (i.e., 1.8–2.0 Gy), the therapeutic index may possibly be improved by increasing the tumor cell kill while reducing side effects to the organs at risk [62]. The success of the recent hypofractionation trials in definitive RT for PCa have provided a rationale for the use of hypofractionation in the postoperative setting where such data are lacking.

However, oncology facilities have been under a lot of pressure due to the COVID-19 pandemic, which has increased the appeal of hypofractionated treatments. Because of its shorter length, there is a lower chance of infection for both staff and patients. Therefore, the international guidelines for treating PCa in response to COVID-19 suggest that modest hypofractionation schedules be used in the adjuvant/salvage scenario as well [63,64]. The year 2024 represented a turning point for the evaluation of hypofractionated treatment in the post-operative setting, as two randomized phase III studies were finally published. RADICALS-RT was designed to compare the efficacy and safety of adjuvant RT after radical prostatectomy versus a policy of observation with early salvage RT for PSA failure. The aim of the study was therefore not to evaluate the effectiveness of a hypofractionated scheme vs. the standard of care. However, at the time of randomization, physicians were free to treat patients using 66 Gy in 33 fractions or 52.5 Gy in 20 fractions, and they included 417 and 217 patients, respectively. With a median follow-up of 5 years, the results of this non-randomized exploratory analysis showed that in the first 2 years, grade 1 to 2 cystitis was reported more frequently among the 66 Gy/33f group (52.5 Gy/20f: 20% vs. 66 Gy/33f: 30%; *p* = 0.04). After 2 years, grade 1 to 2 cystitis was reported in 16% in the 66-Gy group and 9% in the 52.5-Gy group (*p* = 0.08). Other toxic effects were similar in the two groups [46].

The NRG-GU003 trial was the first randomized trials comparing two different RT fractionations in the post-operative setting. The primary objective of NRG-GU003 was to determine whether 62.5 Gy in 25 fractions (2.5 Gy per fraction) resulted in noninferior patient-reported GI and GU symptoms using the Expanded Prostate Cancer Index Composite (EPIC) questionnaire compared with 66.6 Gy in 37 fractions (1.8 Gy per fraction) [47]. Between June 2017 and reaching the target accrual in July 2018, a total of 296 patients were randomized from 93 institutions: 152 to COPORT and 144 to HYPORT. They reported no difference between arms in terms of grade 3 or higher toxicity. Six patients (5%) receiving HYPORT experienced grade 3 cystitis. Only one of these six patients, however, reported urinary function overall as a big problem in the EPIC questionnaire. Patient-reported GI symptoms at 2 years from the completion of RT were not significantly greater when hypofractionation was used. HYPORT was associated with greater patient-reported GI toxic effects compared with COPORT at the completion of RT, but both groups recovered to baseline levels within 6 months. At 2 years, HYPORT was noninferior to COPORT in terms of patient-reported GU or GI toxic effects. Acute side effects after EBRT were usually mild, and it seems that the incidence of side effects does not seem to be associated with NTD2Gy doses.

Indeed, some cases of grade-2 GU side effect were reported in the PRIAMOS 1 trial (NTD2Gy = 58.5 Gy; a/b = 10 Gy), mostly urinary stress incontinence [16]. Furthermore, no grade-2 or higher acute GU toxicity was reported by Lee et al. while treating the prostatic bed to 52.5 Gy in 2.5 Gy per fraction [7]. Cozzarini et al. treated men with 58 Gy at 2.9 Gy per fraction (NTD2 Gy = 62.3 Gy; a/b = 10 Gy) and observed 10% of grade-2 and 2% of grade-3 acute GU toxicity [32]. In the cohort analysis of Wong et al., where 50 patients were treated with a NTD2Gy = 73 Gy (a/b = 10 Gy), an 8% rate of acute grade-2 GU toxicity was reported [25]. Alongi et al. reported four cases (10%) of acute grade-2 GU toxicity when patients received a dose of 74.6 Gy (a/b = 10 Gy) [30].

An increment of 11% in late severe GU toxicity was observed by Cozzarini et al. in men treated with hypofractionation compared to conventional EBRT [32]. Acute grade-2 GU toxicity, in addition to stage pT4 and fractionation schedule, but not NTD2Gy, were identified in multivariate analysis as independent predictive factors associated with severe late GU side effects. Moreover, they reported using a cranio-caudal expansion of 10 mm. The wide margins probably included large bladder volumes that overlapped with the tumor bed, which could account for the high observed risk of GU damage [32]. Concerning acute toxicities, Buwenge et al. showed a statistically significant association between older age (>65) and decreased odds of G ≥ 2 GI acute toxicity (*p*: 0.040) and decreased odds of G ≥ 2 GU acute toxicity (*p*: 0.031) [45]. The 5-year late toxicity-free survival rates for G ≥ 3 GI and GU toxicity were 98.1% and 94.5%, respectively. The only significant correlation found was a reduced risk of late GI toxicity in patients undergoing hypofractionation (*p*: 0.008) [45].

The use of prophylactic irradiation of the pelvic lymphatic areas has been poorly investigated in these series of studies and, if delivered, a conventional fractionation was applied [34,37], with the exception of the Fersino and Koukourakis studies which used doses of 52.5 Gy and 40.7 Gy (2.7 Gy/fr), respectively [15,28]. The effectiveness of postprostatectomy RT can be improved with the addition of ADT and further still with pelvic lymph node RT, although it leads to an increase in GI toxicity. The 5-year biochemical failure-free survival rate from the NRG Oncology/ RTOG 0534 SPPORT trial for men receiving prostate fossa RT with the addition of ADT was 83%, and with the addition of both ADT and pelvic lymph node RT was 89%, compared with 71% for prostate fossa RT alone. Even if the PSA was >0.35 ng/mL, biochemical failure-free survival did not reach a statistical significance. Furthermore, no significant differences in overall survival were reported comparing the three arms [65].

Interestingly, many studies used dose escalation up to 80 NDT2Gy, as required for exclusive treatment of the prostate, while dose escalation studies, using a normofractionated schedule, showed no advantage in administering 70 Gy vs. 66 Gy. This topic is even more interesting today as contemporary imaging (e.g., multiparametric MRI, PET) allows us to detect macroscopic relapses in the prostatic bed. In this setting, some authors have investigated a boost on the macroscopic lesion [66,67], while others have investigated a treatment with the SBRT technique with promising results [68]. The main limitation of the present study is represented by the limited follow-up of many studies, which prevents understanding the long-term effects of hypofractionated treatment. However, Chin et al. retrospectively evaluated 10-yr outcomes of treated men following prostatectomy between 2007 and 2009. Freedom from biochemical failure was associated with presalvage PSA, seminal vesicle invasion and ADT in multivariate analysis.

Despite its retrospective nature, this study provided the longest follow-up of patients treated with hypofractionated salvage RT schedules and confirmed the findings of prior studies suggesting that salvage RT, as soon as the PSA reaches 0.2ng/mL, improves cancer-specific, metastasis-free and overall survival [21].

## 5. Conclusions

The present study is the first systematic review of the literature that includes all the studies published to date, including also the first two randomized phase III studies regarding the use of hypofractionated RT in the treatment of biochemical recurrence after prostatectomy. Although some doubts on late toxic effects remain due to the lack of (still immature) follow-up, hypofractionated treatment has been shown, in retrospective and prospective studies, to be safe, effective, with moderate toxicity and not inferior to conventional RT, with good biochemical control rates. Therefore, a moderate hypofractionated RT should be fully included among the possible treatments (standard of care) to be offered to the patient with biochemical relapse.

## Figures and Tables

**Table 2:** Acute and late toxicity and dose constraints.

Study	Acute Toxicity (%)	Late Toxicity (%)	Constraints
Wong et al. Retrospective (2008) [25]	Acute GU: G1–2: 16; ≥G3: 0 Acute GI: G1–2: 44, ≥G3:0	Late GU: G1–2: 10; ≥G3: 0 Late GI: G1–2: 16; ≥G3: 0	Bladder: <59.5 Gy; Rectum: <20% should receive 60 Gy; Femoral heads: <39 Gy
Kruser et al. Retrospective (2011) [24]	Acute GU: G1–2: 6.4; ≥G3: 0.9 Acute GI: G1–2: 14; ≥G3: 1	Late GU: G1–2: 15; ≥G3: 0 Late GI: G1–2: 4; ≥G3: 0	<20% of the rectal wall receiving 60 Gy; Maximum residual bladder dose < 59.5 Gy; Maximum femoral heads dose < 39 Gy
Koukourakis et al. Phase II (2012) [15]	Acute GU: G1–2: 32.6; ≥G3: 0 Acute GI: ≥G1–2: 81.3; ≥G3: 0	Late GU: ≥G2: 0; ≥G3: 0 Late GI: ≥G2: 0; ≥G3: 0	NR
Ippolito et al. Phase II (2013) [29]	Acute GU: G1–2: 52; ≥G3: 0. Acute GI: G1–G2: 48; ≥G3: 0	Late GU: NR Late GI: NR	Rectum < 25% received 70 Gy; Bladder < 50% received 70 Gy; Small bowel < 2% received 50 Gy; Femoral heads < 10% received 50 Gy
Alongi et al. Retrospective (2013) [30]	Acute GU: G1–2: 5; ≥G3:0 Acute GI: G1–2: 0; ≥G3:0	Late GU: G1–2: 9; ≥G3: 3 Late GI: G1–2: 0; ≥G3:0	Bladder: V60 Gy = 29.9 ± 15.7; Rectum: V60 Gy = 18.9 ± 6.8
Massaccesi et al. Phase II (2013) [31]	Acute GU: G1–2: 9; ≥G3:0 Acute GI: G1–2: 29.7; ≥G3:0	Late GU: NR Late GI: NR	Bladder: <50% ≤ 70 Gy; Rectum: <25% ≤ 70 Gy; Small bowel: <2% ≤ 50 Gy; Femoral heads: <10% ≤ 50 Gy
Cozzarini C. Retrospective (2014) [32]	Acute GU: G1–2: 10; ≥G3: 2 Acute GI: ≥G1–2: 4; ≥G3: 0	Late GU: ≥G3: 46.5 Late GI: ≥G3: 2	NR
Katayama et al. Phase II (2014) [16]	Acute GU: G1–2: 35.9; ≥G3: 0 Acute GI: G1–2: 56.4; ≥G3: 0	Late GU: NR Late GI: NR	Max dose: Anterior rectal wall: 54.3 Gy; Small intestine: 7.4 Gy
Gladwish et al. Phase I/II (2015) [17]	Acute GU: G1–2: 80; ≥G3: 3 Acute GI: G1–2: 0; ≥G3: 0	Late GU: G1–2: 3; ≥G3: 0 Late GI: G1–2: 6; ≥3: 0	NR
Lewis et al. Retrospective (2016) [33]	Acute GU: G1–2: 47; ≥G3: 0 Acute GI: G1–2: 4; ≥G3: 0	Late GU: G1–2: 39; ≥G3: 27 Late GI: G1–2: 41; ≥G3: 3.6	Rectum 50% > 53.8 Gy; 25% > 66.8 Gy; Bladder 50% > 43 Gy; 25% > 62.4 Gy; Small intestine 10% > 44 Gy
Fersino et al. Retrospective (2017) [28]	Acute GU: G1–2: 63; ≥G3: 0 Acute GI: G1–2: 8.8; ≥G3: 0	Late GU: G1–2: 10.4; ≥G3: 1.6 Late GI: G1–2: 8; ≥G3: 0	Rectum: V50 Gy < 45%, V60 Gy < 30%, V65 Gy < 20%, Dmax < 70 Gy; Bladder: V60 Gy < 35%; Femoral heads: D1cm3 < 50 Gy; Abdominal cavity: V20 Gy < 40%, Dmoy < 20 Gy, Dmax < 48 Gy
Macchia et al. Phase II (2017) [34]	Acute GU: G1–2: 42.2; ≥G3: 4 Acute GI: G1–2: 17.7; ≥G3: 0.8	Late GU: G1–2: 17.3; ≥G3: 0 Late GI: G1–2: 1.1; ≥G3: 0	Rectum: 70 GY < 25%; Bladder 70 GY < 50%; Bowel: 50 Gy < 2%; Femoral heads: 50 Gy < 10%
Cuccia et al. Retrospective (2018) [35]	Acute GU: G1–2: 50; ≥G3: 0 Acute GI: G1–2: 54; ≥G3: 0	Late GU: G1–2: 6.6; ≥G3:0 Late GI: G1–2: 5.3; ≥G3: 0	Rectum:V56 Gy ≤ 35%, V60 Gy ≤ 25%; Bladder: V55 Gy ≤ 50%, V60 Gy ≤ 30%; Intestinal cavity: The dose was reduced as much as possible
Tramacere et al. Retrospective (2018) [36]	Acute GU: G1–2: 52.5; ≥G3: 0 Acute GI: G1–2: 34; ≥G3: 0	Late GU: G1–2: 44.6; ≥G3:6 Late GI: G1–2: 6; ≥G3: 1.5	NR
Tandberg et al. Retrospective (2018) [27]	Acute GU: G1–2: 22; ≥G3: 1 Acute GI: G1–2: 5; ≥G3: 0	Late GU: G1–2: 39; ≥G3:11 Late GI: G1–2: 10; ≥G3: 1	NR
Barra et al. Retrospective (2018) [37]	Acute GU: G1–2: 11; ≥G3: 0 Acute GI: G1–2: 5; ≥G3: 0	Late GU: G1–2: 8; ≥G3:3.3 Late GI: G1–2: 8; ≥G3: 0	Rectum: V40 ≤ 43%, V50 ≤ 32%, V65 ≤ 10%; Bladder: V40 ≤ 47%, V55 ≤ 27%, V60 ≤ 14%; Femoral: V20 < 50%; Bowel mean dose: 19.8 Gy; Penile bulb median dose: 39 Gy
Saldi et al. Phase II (2019) [38]	Acute GU: G1–2: 41; ≥G3: 0 Acute GI: G1–2: 46; ≥G3: 0	Late GU: NR Late GI: NR	Bladder: V62 < 50%; Rectum: V39 < 60%, V57 < 40%, V67 < 25%
Ishikawa et al. Retrospective (2020) [20]	Late GI: G2: 0, ≥G3:0 Late GU: G2: 13, ≥G3:0	NR	Rectum: D30 Gy < 70%, V40 Gy < 55%; Bladder: V40 < 50%, V60 Gy < 25%; Penile bulb: V40 < 50%
Leite et al. Phase II 2021) [18]	Acute GU: G2: 11.5, ≥G3:0 Acute GI: G2: 13.1, ≥G3:0	Late GU: G2: 8.2, ≥G3:0 Late GI: G2: 11.5, ≥G3:0	Rectum: D30 Gy < 70%, V40 Gy < 55%; Bladder: V40 < 50%, V60 Gy < 25%; Penile bulb: V40 < 50%
Chin et al. Retrospective (2020) [21]	NR	NR	NR
Wages et al. Phase I/II (2021) [19]	Acute GU: ≥G3: 9.3 Acute GI: ≥G3: 3.12	NR	NR
Valero et al. Retrospective (2021) [39]	Acute GU: G2: 8, ≥G3: 0 Acute GI: G2: 3.5, ≥G3: 0	Late GU: G3: 1 Late GI: G2: 2, ≥G3: 0	Rectum: Dmean < 44.5 Gy V60.9 < 44.7 Gy 1459 V67.4, V65.2 < 15% - V60.9 < 20% -V40 < 60%; Bladder: Dmean < 44.5 Gy - V67.4 < 15% - V65.2 < 25% - V60.9 < 30% - V47.5 < 50%; Penile bulb: Dmean < 44.7 Gy; Femoral heads: V44.7 < 10%
Franzese et al. Retrospective (2021) [40]	Acute GU: ≥G3: 2.7 Acute GI: ≥G3: 1.6	NR	Rectum: D60 Gy < 20%, D55 Gy < 30%, D46 Gy < 40%, D42 Gy < 45%, D37 Gy < 50%; Bladder: Dmax < 66.9 Gy, D60 Gy < 20%, D53 Gy < 40%, D48 Gy < 50%, D43 Gy < 55%, D39 Gy < 60%, D29 Gy < 80%; Femoral heads: Dmax < 59 Gy, D50 Gy < 5%
Murgic et al. Retrospective (2021) [22]	NR	Late GU: ≥G3: 8	NR
Viani et al. Prospective (2022) [41]	Acute GU: G1: 24.5%; G2: 6.6%; ≥G3: 0 Acute GI: G1: 11.1%; G2: 2.2%; ≥G3: 0	Late GU: G1: 20%; G2: 4.4%; ≥G3: 0 Late GI: G1: 33.3%; G2: 2.2%; ≥G3: 0	Rectum: D60 Gy < 20%; Bladder: NR; Penile bulb: NR
Ferrera et al. Retrospective (2022) [42]	Acute GU: G1–2: 47.9, ≥G3: 0 Acute GI: G1–2: 44.3 ≥G3: 0	Late GU: G1–2: 10.4, ≥G3: 1.5 Late GI: G1–2: 9.2, ≥G3: 0	Rectum: V56 Gy ≤ 35% and V60 Gy ≤ 25%; Bladder: V55 Gy ≤ 50% and V60 Gy ≤ 30% to 35%; Intestinal cavity: Dose reduced as much as possible
Nicosia, L et al. Retrospective (2022) [26]	Acute GU: G1–2: 36, ≥G3: 3.2	NR	Bladder: V60 Gy < 35%; Rectum: V50 Gy < 45%, V60 Gy < 30%, V65 Gy < 20%, Dmax < 70 Gy; Femoral heads: D1cc < 50 Gy; Bowels: V20 Gy < 40%, Dmean < 20 Gy Dmax < 48 Gy
Moll et al. Retrospective (2022) [43]	Acute GU: G1–2: 9, ≥G3:0 Acute GI: G1–2: 75, ≥G3:0	Late GU: G1: 86, G2: 12, ≥G3:2 Late GI: G1: 92, G2: 8, ≥G3:1	Rectum: D60 Gy < 20%, D55 Gy < 30%,D46 Gy < 40%, D42 Gy < 45%, D37 Gy < 50%; Bladder: Dmax < 66.9 Gy, D60 Gy < 20%, D53 Gy < 40%, D48 Gy < 50%, D43 Gy < 55%, D39 Gy < 60%, D29 Gy < 80%; Femoral heads: Dmax < 59 Gy, D50 Gy < 5%
Dubinsky et al. Prospective (2023) [44]	Acute GI: G2: 24, G3: 2 Acute GU: G2: 10, G3: 0	Late GI: G2: 9, Late GU: G2: 16	NR
Buwenge et al. Retrospective (2023) [45]	NR	NR	Rectum: Dmean < 44.5 Gy V60.9< 44.7 Gy 1459 V67.4, V65.2 < 15% - V60.9 < 20% -V40 < 60%; Bladder: Dmean < 44.5 Gy - V67.4 < 15% - V65.2 < 25% - V60.9 < 30% - V47.5 < 50%; Penile bulb: Dmean < 44.7 Gy; Femoral heads: V44.7 < 10%
Petersen et al. Phase III (2023) [46]	Acute GI: G1–2: ≥G3: 6 Acute GU: ≥G3: 9	Late GI: ≥G3: 1 Late GU: ≥G3: 6	Bladder: 40 Gy < 80%, 48 Gy < 50%; Rectum: 24 GY < 80%, 32 GY < 70%, 40 GY < 60 Gy, 48 Gy < 50%, 52.5 GY < 30%
Castelluccia et al. Retrospective (2024) [23]	NR	Late GI: ≥G3: 1.8 Late GU: ≥G3: 8	Rectum: D60 Gy < 20%, D55 Gy < 30%, D46 Gy < 40%, D42 Gy < 45%, D37 Gy < 50%; Bladder: Dmax < 66.9 Gy, D60 Gy < 20%, D53 Gy < 40%, D48 Gy < 50%, D43 Gy < 55%, D39 Gy < 60%, D29 Gy < 80%; Femoral heads: Dmax < 59 Gy, D50 Gy < 5%
Buyyounouski et al. Phase III (2024) [47]		Late GI: ≥G3: 13 Late GU: ≥G3: 7	Rectum: D60 Gy < 20%, D55 Gy < 30%, D46 Gy < 40%, D42 Gy < 45%, D37 Gy < 50%; Bladder: Dmax < 66.9 Gy, D60 Gy < 20%, D53 Gy < 40%, D48 Gy < 50%, D43 Gy < 55%, D39 Gy < 60%, D29 Gy < 80%; Femoral heads: Dmax < 59 Gy, D50 Gy < 5%

Abbreviations: G: grade; GU: genitourinary; GI: gastro-intestinal; NR: not reported; Gy: Gray; D: dose; V: volume; Dmax: maximal dose; Dmean: medium dose.

## Data Availability

Not applicable.
